# Small RNAs Worm Up Transgenerational Epigenetics Research

**DOI:** 10.3390/dna1020005

**Published:** 2021-09-29

**Authors:** Alla Grishok

**Affiliations:** Department of Biochemistry, BU Genome Science Institute, Boston University School of Medicine, 72 E. Concord St. K422, Boston, MA 02118, USA;

**Keywords:** transgenerational epigenetic inheritance (TEI), *C. elegans*, double-stranded RNA (dsRNA), RNA interference (RNAi), small interfering RNAs (siRNAs), Piwi-interacting RNAs (piRNAs), gene silencing, adaptation

## Abstract

DNA is central to the propagation and evolution of most living organisms due to the essential process of its self-replication. Yet it also encodes factors that permit epigenetic (not included in DNA sequence) flow of information from parents to their offspring and beyond. The known mechanisms of epigenetic inheritance include chemical modifications of DNA and chromatin, as well as regulatory RNAs. All these factors can modulate gene expression programs in the ensuing generations. The nematode *Caenorhabditis elegans* is recognized as a pioneer organism in transgenerational epigenetic inheritance research. Recent advances in *C. elegans* epigenetics include the discoveries of control mechanisms that limit the duration of RNA-based epigenetic inheritance, periodic DNA motifs that counteract epigenetic silencing establishment, new mechanistic insights into epigenetic inheritance carried by sperm, and the tantalizing examples of inheritance of sensory experiences. This review aims to highlight new findings in epigenetics research in *C. elegans* with the main focus on transgenerational epigenetic phenomena dependent on small RNAs.

## 1. Introduction

**Figure 1.**
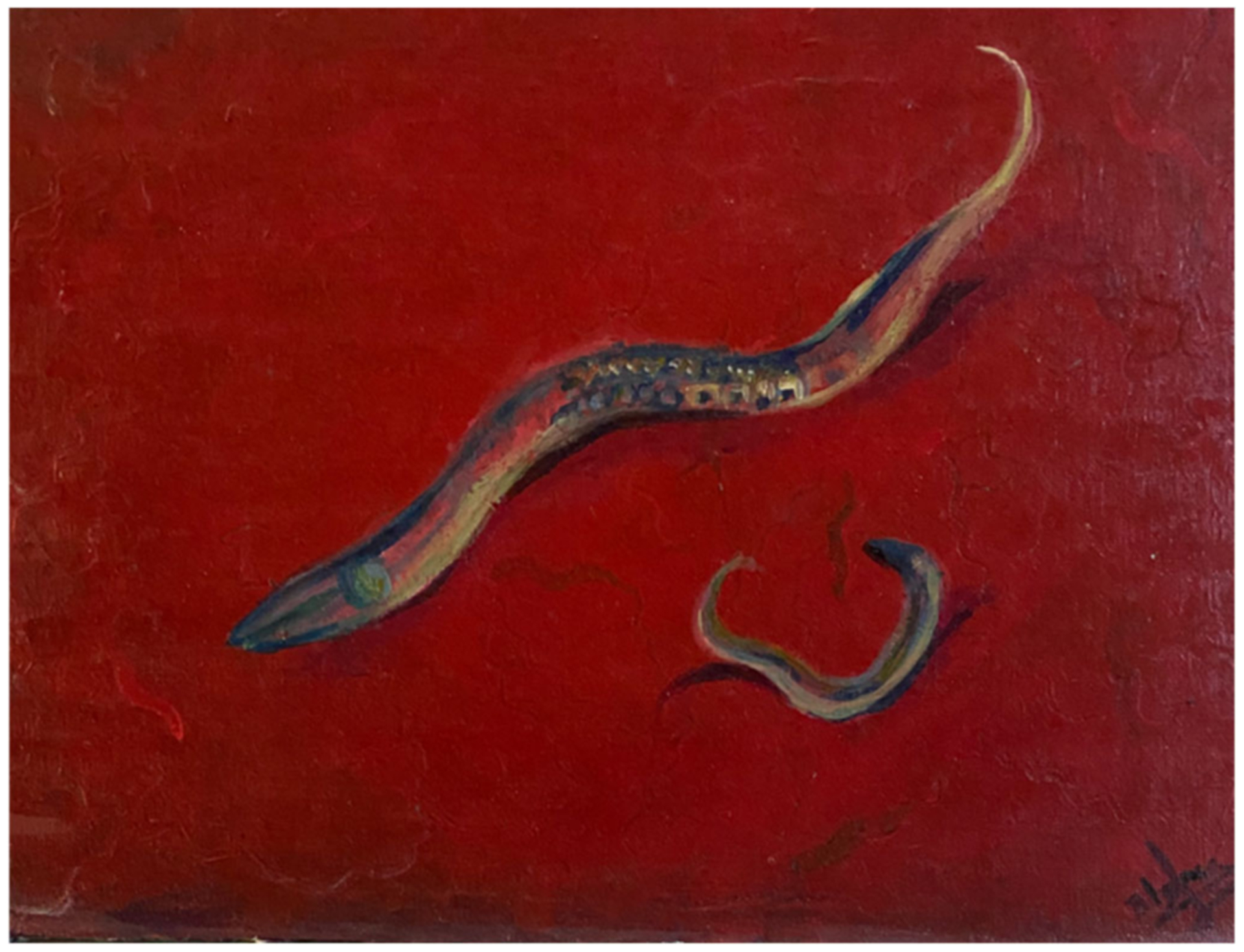
Artistic representation of the adult *Caenorhabditis elegans* hermaphrodite and a young larva crawling on a plate. Image of the oil painting “Red” by Blanca Craven-Bartle (2015).

Numerous discoveries in basic science were facilitated by the model organism *Caenorhabditis elegans* (reviewed in [1]) (Figure 1).

*C. elegans* is especially rich in small regulatory RNAs that control most aspects of its development, lifespan, and reproduction. The best-known class of small RNAs, microRNA (miRNA), was discovered through genetic analyses of cell lineage in *C. elegans* that resulted in the identification of the founding members of the miRNA family: *lin-4* in 1993 [2,3] and *let-7* in 2000 [4,5], by Ambros, Ruvkun, and their colleagues. The significance of miRNAs in medicine through their use as biomarkers, drug targets, or RNA drugs is beyond dispute (reviewed in [6]), and it is hard to imagine the loss to society had miRNAs not been discovered.

One of the most fascinating areas of *C. elegans* research is the epigenetic inheritance of small RNAs and their potential roles in shaping genome architecture and mediating heritable adaptive responses to environmental challenges. The connection between the human diet and epigenetic adaptation to existing conditions via heritable RNA transmission in sperm (reviewed in [7]) underscores the importance of lessons learned from *C. elegans*.

This manuscript provides an overview of key findings that may interest a wide scientific audience, including the highlights from the Genetics Society of America 23rd International *C. elegans* Conference, which was held virtually on 21–24 June 2021. Detailed descriptions of the field targeting specialists can be found elsewhere, for example [8–13].

## 2. *C. elegans* Model Systems for Studying Transgenerational Epigenetic Inheritance (TEI)

**Figure 2.**
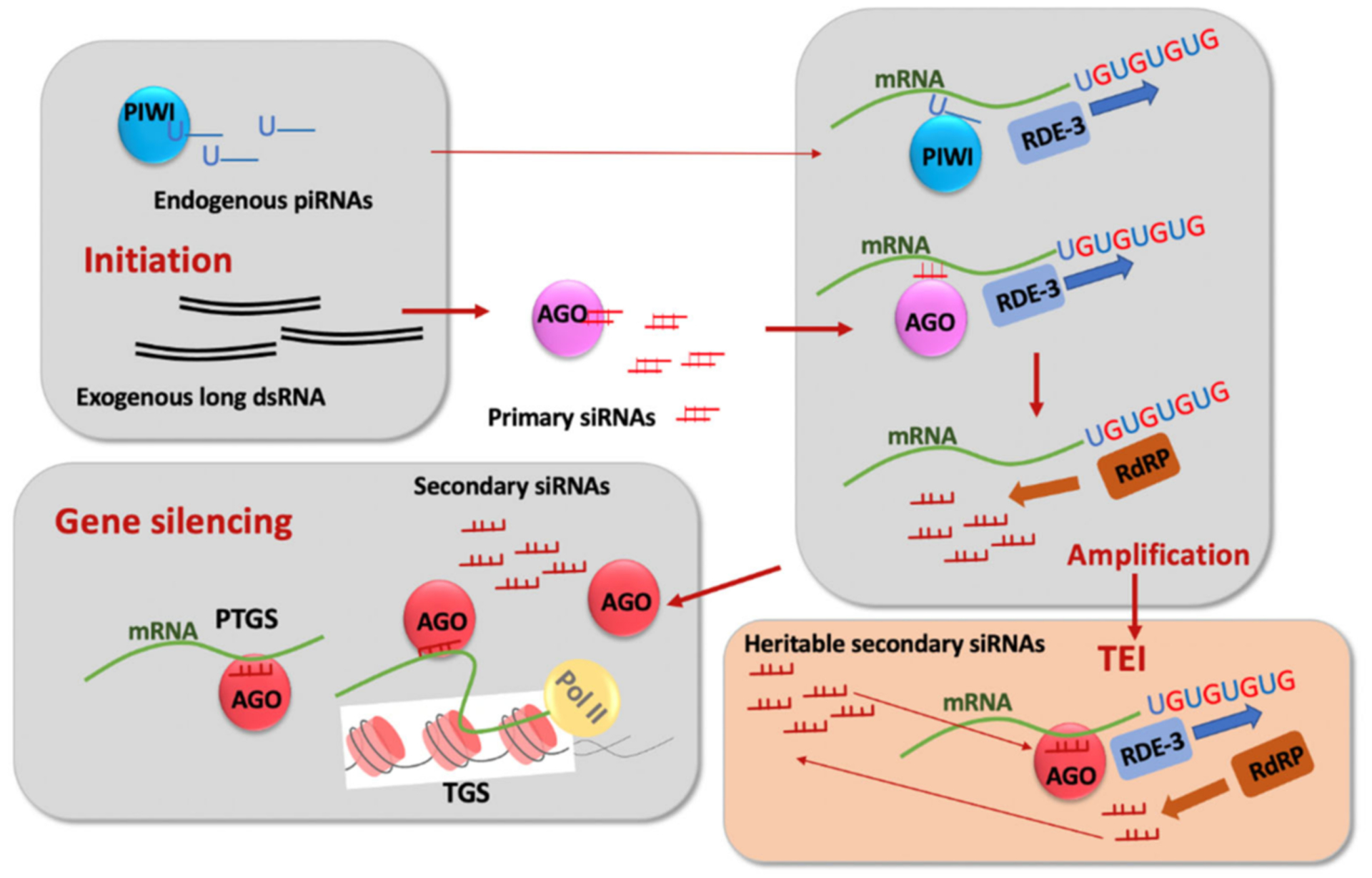
Illustration of the transgenerational epigenetic inheritance steps in the *C. elegans* germline: initiation via double-stranded RNA (dsRNA), which is processed by the Dicer complex into primary small interfering RNAs (siRNAs) bound by Argonaute (AGO) or via Piwi-interacting RNA (piRNA) bound to PIWI; amplification of siRNAs on mRNA templates bound by AGO or PIWI, which includes non-templated UGn addition by the nucleotidyltransferase RDE-3 followed by secondary siRNA production by RNA-dependent RNA polymerase (RdRP). Secondary siRNAs are bound by AGO that are distinct from those binding primary RNAs. Secondary siRNA/AGO complexes induce either post-transcriptional (PTGS) or transcriptional (TGS) gene silencing. Secondary siRNAs are thought to be heritable and capable of initiating the siRNA amplification cycle in the germline of progeny.

The first indication of the potent role of RNA molecules in inhibiting the expression of *C. elegans* genes came from applications of antisense RNA technology to nematodes [14,15]. This was followed by the seminal discovery of double-stranded RNA (dsRNA) [16] (Figure 2) as an initiator/intermediate in the silencing process termed RNA interference (RNAi) [16,17] and the identification of small interfering RNAs (siRNAs) (Figure 2) as key players in silencing induced by dsRNA in various organisms [18].

Although RNAi of genes expressed in the soma persisted in first-generation progeny (F1) of treated animals and was not inherited further [16], it was soon realized that the dsRNA-induced phenotypes mimicking mutations in germline-expressed genes could persist longer than the F1 generation and could be transmitted via factors not linked to the target DNA locus and distinct from dsRNA (likely siRNAs) [19]. Subsequent research identified the RNA-dependent RNA polymerase (RdRP)-based siRNA amplification mechanism using mRNAs as templates [20–24] (Figure 2). The outstanding question of how RdRPs find their appropriate target RNAs has recently been answered by the discovery of 3′ tags consisting of non-templated UG additions that attract RdRPs [25–27] (Figure 2).Since the deposition of UG tags required for siRNA regeneration is ultimately initiated by siRNAs, this system can lead to the potentially indefinite perpetuation of siRNA-induced gene silencing on post-transcriptional or co-transcriptional levels [26] (Figure 2).

Since most germline-expressed genes are essential for viability, their silencing compromises the production of gravid progeny, thus hampering inheritance studies. Therefore, new systems where silencing could be monitored for many generations were needed. In one such system, which is based on the silencing of the *oma-1* gene containing a conditional-lethal mutation, and which monitors animal survival rather than death, the effect of the initial dsRNA exposure was shown to persist for four generations [28]. Other systems were made possible by technical advances in transgenic technology that allowed stable expression of transgenic arrays in the *C. elegans* germline [29]. This allowed investigators to monitor germline GFP transgene silencing in the progeny of animals exposed to dsRNA, which was shown to last up to twenty generations with selection [30,31]. When the single-copy controlled genomic integration of transgenes was developed [32,33], it further facilitated Transgenerational Epigenetic Inheritance (TEI) research [34–36]. Most, but not all [37], subsequent studies researching mechanisms of TEI in *C. elegans* utilized the *oma-1* and/or germline GFP readouts.

## 3. RNA-Based and Chromatin-Based Epigenetic Silencing and Their Connections

In the last 10–15 years, TEI phenomena have been described in the context of gene silencing induced by endogenous Piwi-interacting RNAs (piRNAs) (Figure 2), and a broad term to describe long-lasting silencing has been introduced: RNA-induced epigenetic silencing (RNAe) [36,38,39]. The targets used to monitor the persistence of piRNA-induced silencing are usually single copy germline-expressed GFP-based sensors containing sequences complementary to some endogenous piRNAs. The persistence of piRNA-induced RNAe, similarly to dsRNA-induced RNAe, relies on the amplification cycle of siRNAs [36,38–40] (Figure 2). In addition to siRNA amplification, epigenetic inheritance of the compacted chromatin state is thought to play a role in various TEI phenomena [34,36,38]. In some examples of TEI, maintenance of silencing persisted apparently indefinitely [36,38]. Until very recently, the dependence of TEI on small RNAs and the silencing-associated chromatin marks [34,36,38,41,42] has been interpreted in terms of an RNA-dependent initiation step followed by a chromatin-based maintenance step [34,36,42]. However, recent studies showed that chromatin regulators may act as early as in the P0 (parents exposed to dsRNA) generation of dsRNA-induced TEI, whereas siRNA amplification sustains TEI maintenance [35,43]. Notably, dsRNA-induced silencing of repetitive transgenes expressed in somatic tissues occurs at the transcriptional level in the F1 generation of treated worms [44], and the somatic nuclear RNAi pathway is required for gene silencing in the soma of F1 larvae [45]. However, transcriptional silencing of somatic genes does not display TEI.

Both maternal and paternal inheritance of RNA-induced epigenetic silencing is usually observed in *C. elegans* [19,28,36,46]. A variety of RNA- and protein-rich granules, which are not bound by membranes, exist in the *C. elegans* germline. These granules contain mRNA and proteins implicated in the biogenesis and function of small RNAs, including those mediating TEI (reviewed in [47]). At least some of these granules, such as P-granules, are present in oocytes [48] and therefore could harbor heritable siRNA generated through the amplification cycle (see Section 2). The most recent work by Ketting and colleagues determined that mature sperm does not inherit granules that are common between the male and hermaphrodite germlines and described a novel sperm-specific condensate—PEI (paternal epigenetic inheritance) granule—named after its resident protein PEI-1 [49].Importantly, the authors identify *pei-1*-like genes in humans, which points to the possible conservation of paternal epigenetic inheritance mechanisms.

## 4. Permanent and Limited Forms of TEI and Their Genetic Control

In *C. elegans*, a process of indefinite silencing of repetitive transgenes designed to be expressed in the germline has been described by Kelly and co-authors in 1997 [50]. It was shown to be related to but distinct from the dsRNA-induced gene silencing [51]. The use of single-copy integrated transgenes for TEI research is convenient but at the same time begs the question of its relevance to the understanding of the epigenetic mechanisms involved in endogenous gene regulation. The piRNA-induced RNAe in particular has been likened to epigenetic silencing of parasitic elements that must be inactivated indefinitely [36,39,40,52].

An interesting case of mating-induced silencing of a single copy transgene, which fortuitously contained DNA sequences complementary to endogenous piRNAs, has been reported recently by Jose and colleagues [53]. In this system, the transgene inherited paternally was subject to silencing by maternal piRNAs that lasted apparently indefinitely (>300 generations) and required siRNA amplification machinery in each generation for its propagation [53]. This case illustrates the efficiency of maternal piRNAs in inducing silencing of the “foreign” DNA inherited through paternal sperm. Importantly, when the mothers also expressed the transgene, mating-induced silencing did not occur [53] underscoring the discrimination between “self” and “nonself” proposed by Mello and colleagues [36]. This example of TEI in *C. elegans* resembles the phenomenon in *Drosophila* where maternal piRNAs silence paternally-inherited transposons [54].

The mutants that disrupt RNAe generally display a mortal germline phenotype (Mrt)—a progressive sterility that increases over multiple generations [55]. Both the de-repression of normally silenced repetitive elements [52] and the inappropriate silencing of essential endogenous genes, such as histones [56,57], were correlated with the Mrt phenotype. A very recent study by Fire and co-workers identified enhanced production of small RNAs antisense to rRNA as a likely cause of the Mrt phenotypes in animals lacking piRNAs [58]. Guang and colleagues characterized this new class of antisense ribosomal siRNAs (risiRNAs) earlier and had shown that they inhibit rRNA expression via the nuclear RNAi pathway (reviewed in [59]). Importantly, copy-number amplification of rDNA delayed the onset of sterility in piRNA-depleted worms supporting the causal role of impaired ribosome function in the Mrt phenotype [58].

Model systems displaying limited TEI that lasts for 6–7 generations are more suitable for studying adaptive and dynamic epigenetic phenomena. The important question in such systems is: what determines the escape from TEI after several generations? Genetic screens seeking mutants allowing longer TEI revealed the existence of control mechanisms limiting its duration [60]. This finding is conceptually significant because it demonstrates that organisms might not only allow a certain degree of environmentally-induced epigenetic change but also control its duration. The inhibitors of TEI include both chromatin regulators, such as a histone methyltransferase MET-2 [61] and a chromatin-binding protein CEC-9/HERI-1 [60], and RNA-based mechanisms [60]. Intriguingly, piRNAs are capable of both initiating TEI and suppressing its maintenance [62,63], although it is not clear whether the latter effect is direct. Notably, although the siRNA amplification system is unique to nematodes, piRNAs, as well as their RdRP-independent amplification machinery, are present in the germlines of all animals [64].

## 5. Coordination between Gene Silencing in the Soma and Germline; Who Is the Messenger?

**Figure 3.**
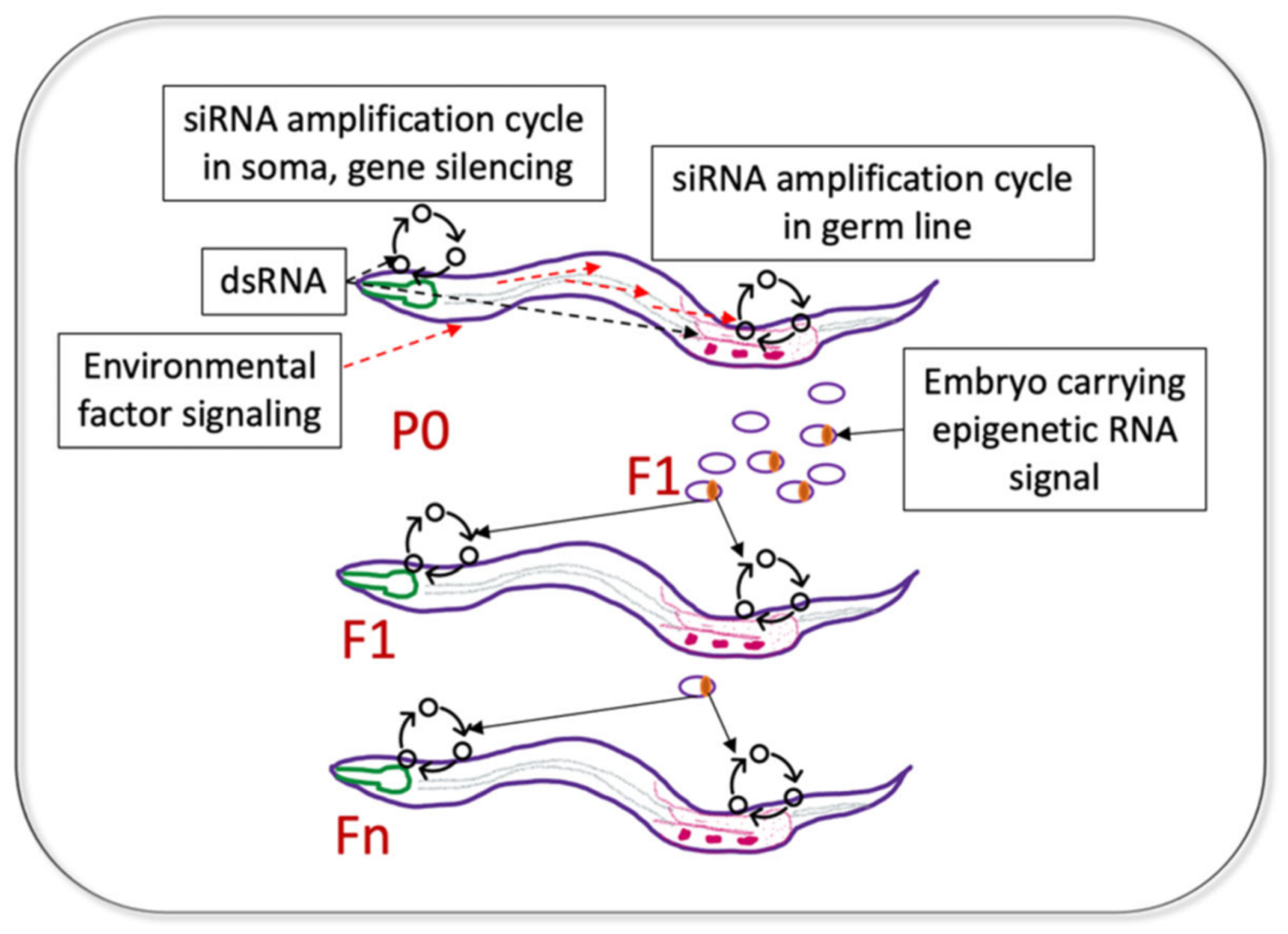
Possible mechanisms of heritable transmission of adaptive responses for genes with dual germline and soma expression, based on published work. dsRNA segments found in bacterial ncRNA can initiate a physiological response in tissues expressing mRNAs complementary to dsRNA (siRNA amplification in soma, gene silencing). At the same time, the ingested dsRNA can travel to the germline and initiate an siRNA amplification cycle on the same mRNAs. The heritable epigenetic RNA signal (likely secondary siRNA) is deposited in the F1 embryos; when these siRNAs are distributed to tissues expressing their mRNA targets, they initiate new siRNA amplification cycles. The initiation of gene silencing in the target somatic tissue (e.g., neuron) leads to the phenotypic manifestation of the adaptive response in F1 (e.g., pathogen avoidance), whereas amplification of siRNAs on the same mRNAs expressed in the germline leads to propagation of the adaptive responses through generations. Note that the environmental signal sensed by the P0 worms does not need to be dsRNA, but this signal has to ultimately induce the siRNA amplification cycle in the germline.

The inheritance of gene silencing by the immediate (F1) progeny of dsRNA-exposed worms was recognized early [16,19]. The F1 generation showed the strongest phenocopy of the corresponding DNA mutations in a number of germline-expressed and soma-expressed genes [16]. Remarkably, their progeny (the F2 generation) completely lost the dsRNA-induced somatic phenotypes [16]. However, subsequent work identified certain somatic genes whose silencing persisted in successive generations [30] (Figure 3).

Now, there are reasons to think that such genes are expressed in both the germline and some somatic tissues. This dual expression requirement for the RNA-induced TEI of somatic gene silencing has been postulated by Minkina and Hunter who studied TEI of the endogenous *sid-1* gene [37]. In this example, both germline and somatic silencing of endogenous *sid-1* were triggered by a repetitive germline-expressed transgene with homology to the *sid-1* locus and were associated with the siRNAs antisense to the *sid-1* coding region. Whereas *sid-1* silencing in the germline persisted for 8–13 generations, somatic *sid-1* silencing was observed for four generations and was dependent on the germline-expressed factors binding heritable amplified small RNAs. Thus, for genes with dual germline and soma expression, a heritable transmission of a favorable adaptive somatic phenotype (e.g., heat shock resistance, pathogen avoidance) is mechanistically possible in *C. elegans* [37] (Figure 3).

An important feature of the dsRNA-induced silencing in *C. elegans* is its systemic nature [16]. This is facilitated by the expression of the dsRNA-selective dsRNA-gated channel, SID-1 [65,66]. Thus, dsRNA species expressed through transgenes driven by tissue-specific promoters were used to silence homologous genes in the other tissues [67]. Notably, a GFP-specific dsRNA expressed in neurons triggered a persistent and self-sustaining silencing of a germline GFP transgene [68]. Therefore, it is possible that the communication between the somatic and germline tissues via endogenously produced dsRNA species exists in nematodes and contributes to adaptive TEI (Figure 3). The conservation of the SID-1 protein in mammals [65] suggests a possibility of dsRNA transport in higher organisms as well.

## 6. Sensory Experiences Communicated to the Germ Line and Transmitted Transgenerationally

There is a growing list of environmental conditions, including starvation [69], dauer diapause [70], and pathogen exposure [71–73], which elicit gene expression changes, developmental program variations, and adaptive behaviors that persist in multiple generations. Based on genetic analyses in different experimental systems, the heritable biological responses to environmental changes appear to rely both on dsRNA transport from the soma to the germline [73] and on germline TEI driven by piRNAs and/or siRNA amplification [69,70,72,73]. Most recently, Zaslaver and colleagues designed *C. elegans* training experiments inducing an associative memory between an odorant and starvation, which was monitored via nuclear translocation of a global stress response factor upon memory reactivation [74]. Remarkably, this association persisted in the progeny of trained worms for two generations and required both RNAi and chromatin regulators [74].

The best mechanistically understood phenomenon has been described by Murphy and colleagues [73] who investigated a specific learning behavior—avoidance of *Pseudomonas aeruginosa* (PA14)—that persisted for four generations [72]. Remarkably, it was found that *C. elegans* interpreted a specific PA14 ncRNA transcript as a pathogen signal and built an RNAi-based response to avoid it [73]. The components of this response included the dsRNA transport machinery as well as the piRNA-based germline TEI system. The authors proposed that the complementarity between a section of the bacterial P11 RNA and the mRNA coding for neuronal MACO-1 protein induced an RNAi response and caused MACO-1 downregulation. This, in turn, led to the activation of the TGF- ligand DAF-7 in a specific neuron, which ultimately caused avoidance behavior. Interestingly, *maco-1* mRNA expression has been detected in the germline [75,76], in addition to the neurons. Therefore, the biologically-relevant heritable response of *C. elegans* to PA14-derived ncRNA that involves *maco-1* inhibition is reminiscent of the artificial TEI system where the connection between the somatic and germline silencing of *sid-1* has been elucidated ([37] see Section 5) (Figure 3).

## 7. Epigenetics of Holocentric Centromeres

**Figure 4.**
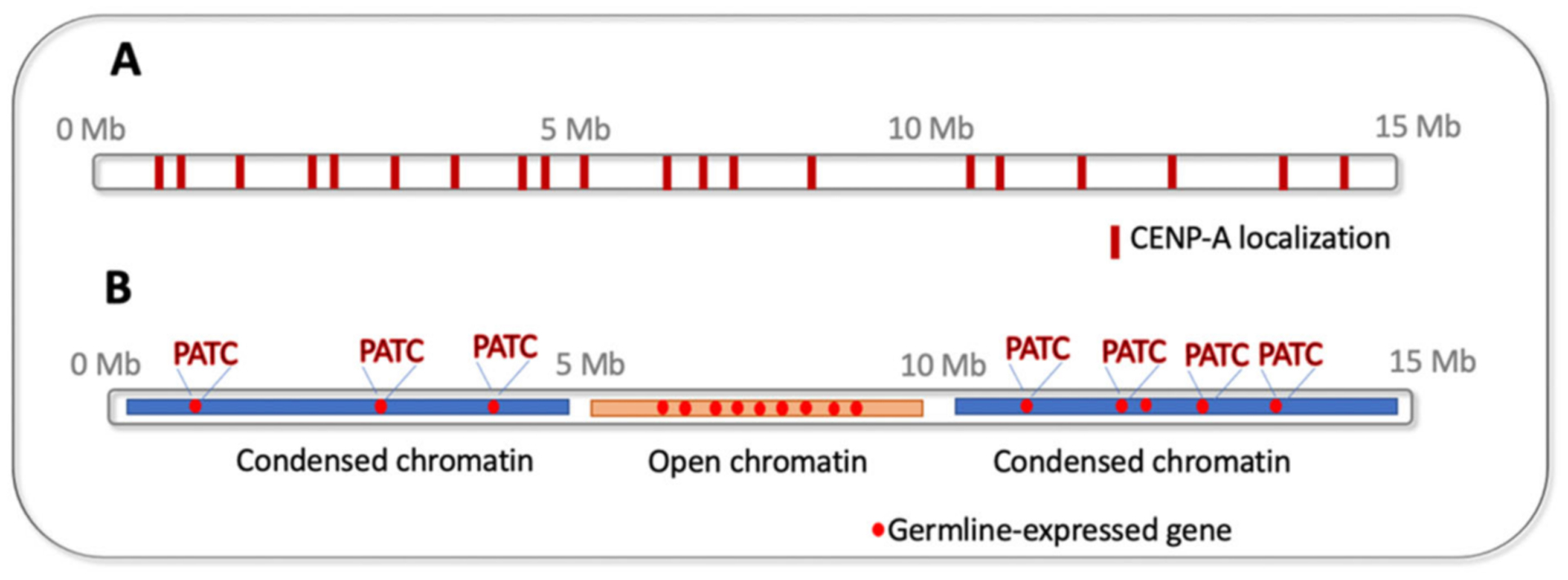
Epigenetic features of *C. elegans* chromosomes (an autosome schematic is shown): (**A**) distribution of CENP-A; (**B**) open chromatin on autosome centers and condensed chromatin on autosome arms, germline-expressed genes located on autosome arms contain 10-base pair periodic An/Tn-clusters (PATCs) that facilitate their activation.

In contrast to the dynamic adaptive TEI phenomena discussed in Sections 2–6, there are also very stable epigenetic processes ensuring proper genome maintenance across generations. These include the epigenetic inheritance of the centromeric chromatin positioning. Centromeres are specialized structures on chromosomes that are required for proper microtubule attachment and faithful chromosome segregation. They are marked by the centromere-specific histone variant protein, CENP-A. CENP-A localization on daughter DNA strands is informed by its position on the maternal templates (reviewed in [77]). In most organisms, centromeric chromatin is concentrated in one chromosomal location. The *C. elegans* chromosomes are holocentric, so CENP-A is distributed in numerous foci along the chromosomes (reviewed in [78]) (Figure 4a). Moreover, in the *C. elegans* germline there is a disruption of the templated inheritance of CENP-A during meiosis [79]. This begs the question of how the correct chromosomal locations are marked for de novo CENP-A deposition. Although the nature of the positioning mark is still not clear, Steiner and colleagues determined that the de novo placement of CENP-A occurs during a strict developmental window preceding the first embryonic division of the zygote [80]. This process is dependent on the N-terminal tail of CENP-A [80,81]. Surprisingly, the N-terminal tail is dispensable for mitotic divisions during late embryogenesis, larval development, and germline proliferation [80]. Thus, there are distinct steps of initiation and maintenance governing the epigenetic inheritance of CENP-A between generations in *C. elegans*.

What could serve as a signal for the de novo CENP-A placement in the zygote? Earlier studies from Desai, Strome, and colleagues suggested that the memory of germline transcription is key [82]. Importantly, there are worm antisense siRNAs produced by RdRPs on germline-expressed mRNA templates that are distinct from the silencing siRNAs [83]. They are bound to the Argonaute CSR-1, which is present both in the cytoplasm and the nucleus [84,85]. The nuclear CSR-1 complex is thought to mark genomic regions repelling the silencing chromatin marks [84,86,87], which correlate with CENP-A placement [82,86]. The mechanism of this is not clear. If this scenario is correct, then CENP-A incorporation should proceed by default. There is also the possibility of both repelling and attracting marks guiding the de novo CENP-A pattern establishment.

## 8. DNA “Watermarks” Allowing Gene Expression in Silenced Chromatin Environment

In *C. elegans*, “silencing” chromatin marks are enriched on autosome arms [88] whereas the essential genes, including most germline genes, are located at the centers of autosomes [89] (Figure 4b). Therefore, it is thought that the chromosome location (i.e., active or closed chromatin environment) determines whether the gene is expressed in the germline or not. Surprisingly, this is not the whole story. Frøkjær-Jensen and co-authors discovered periodic DNA sequence patterns, namely 10-base pair periodic An/Tn-clusters (PATCs), which are correlated with germline expression of the germline genes located on autosome arms [90] (Figure 4b). Moreover, the inclusion of such sequences on the repetitive transgenic arrays allowed their germline expression. Although the mechanism of this phenomenon is enigmatic, and the *Caenorhabditis*-specific PATCs periodicity is not seen in more distantly related species [90], there might be other periodic DNA marks that are biologically relevant in other organisms.

## 9. Concluding Remarks

The recent literature reviewed here underscores the value of basic epigenetics research in a nematode model. More importantly, recent discoveries pose tantalizing questions for future inquiries. The importance of small RNAs in transgenerational transgene silencing and in aberrant progressive silencing of endogenous genes, which underlies the Mrt phenotype, is well documented. However, the mechanistic links between small RNAs and histone-modifying complexes participating in these phenomena are poorly understood and should be investigated in the future. There is still a debate on whether the inappropriate silencing of just one category of repetitive genes, such as the rDNA repeats or histone gene loci, is responsible for the Mrt phenotype of animals lacking piRNAs. It is likely that the silencing of both types of targets contributes to the Mrt sterility. Moreover, it was shown that the Mrt phenotype can be suppressed by insulin signaling pathway mutants [91]. It is not clear whether the insulin signaling pathway regulates small RNAs matching histone genes, rDNA repeats, or something else. It is curious that insulin signaling mutant backgrounds allow a more robust response to experimental RNAi [92] further underscoring the connection to small RNA regulation.

Although the systemic nature of *C. elegans* RNAi has been known for a long time, and the possibility of silencing information transport from one tissue to another has been shown using transgenes, examples of endogenous RNAi transport are lacking. A recent implication of the dsRNA transport machinery in pathogen avoidance behavior [73] brings hope that the sequence of the dsRNA carrying the avoidance signal and other natural dsRNAs will be uncovered soon.

The participation of small RNAs in the memory of sensory experiences is largely concluded from the experiments with the mutants in various RNAi-related pathways. Since multiple distinct pathways are often involved, it is not easy to imagine specific mechanistic scenarios behind the phenomenology. The challenges in future work will be in dissecting the order of RNAi components’ action and in distinguishing between direct and indirect involvement of small RNAs.

The “licensing” role of small RNAs that do not cause gene silencing but rather protect from it remains enigmatic, especially with regards to nuclear phenomena, despite much progress in the field. Specifically, the mechanistic connection or cooperation between the nuclear CSR-1 pathway and the molecular marks of active chromatin must be understood. Finally, there is still the possibility that PATCs DNA sequences act through RNA intermediates, perhaps small RNAs, to facilitate germline expression of genes located in a closed chromatin environment.
